# The impact of resistance exercise range of motion on the magnitude of upper-body post-activation performance enhancement

**DOI:** 10.1186/s13102-022-00519-w

**Published:** 2022-07-07

**Authors:** Michał Krzysztofik, Robert Trybulski, Bartosz Trąbka, Dawid Perenc, Konrad Łuszcz, Adam Zajac, Dan Iulian Alexe, Tatiana Dobrescu, Cristina Elena Moraru

**Affiliations:** 1 Institute of Sport Sciences, The Jerzy Kukuczka Academy of Physical Education in Katowice, Mikołowska 72A str., 40-065 Katowice, Poland; 2 Provita Zory Medical Center, 44-240 Zory, Poland; 3 Department of Medical Sciences, The Wojciech Korfanty School of Economics, 40-659 Katowice, Poland; 4 Faculty of Physical Education, Gdansk University of Physical Education and Sport, 80-336 Gdansk, Poland; 5 Faculty of Electrical Engineering, Silesian University of Technology, 44-100 Gliwice, Poland; 6 Department of Sports Training, The Jerzy Kukuczka Academy of Physical Education in Katowice, 40-065 Katowice, Poland; 7 Faculty of Movement, Sports and Health Sciences, Vasile Alecsandri University of Bacău, 600115 Bacau, Romania; 8 Faculty of Physical Education and Sport, Alexandru Ioan Cuza University of Iasi, 700506 Iasi, Romania

**Keywords:** Bench press, Bench press throw, Conditioning activity, PAPE, PAP, Post-activation potentiation

## Abstract

**Background:**

Various studies have used different exercise protocols as post-activation performance enhancement (PAPE) stimulus; however, little attention has been given to the effects of exercise range of motion on the PAPE effect and subsequent performance enhancement. This study aimed to compare the PAPE responses induced by the bench press performed with different ranges of motion on subsequent bench press throw performance.

**Methods:**

Ten resistance-trained males (age: 26 ± 3 years; body mass: 93.2 ± 9.4 kg; height: 181 ± 6 cm; experience in resistance training: 6.3 ± 2.4 years; relative bench press one-repetition maximum (1RM) 1.54 ± 0.2 kg/body mass) performed four experimental sessions consisting of a single set of the bench press at 80%1RM until mean barbell velocity dropped by 10% as the conditioning activity (CA) with a (1) standard, (2) cambered, (3) and reversed cambered barbell or a control condition in which the participants did not perform any CA. To assess the PAPE effect, single-sets of 2 repetitions of the bench press throw at 30%1RM were performed before and after the CA at the following time points: 2, 4, 6, 8, 10 min.

**Results:**

The two-way ANOVA (4 conditions × 2time points) showed a significant interaction for peak power (p < 0.001; η^2^ = 0.556) and peak velocity (p = 0.001; η^2^ = 0.457). The standard barbell bench press CA led to the greatest performance enhancement in peak power (p = 0.001; ES = 0.54) and in peak velocity (p = 0.002; ES = 0.71) within the examined conditions.

**Conclusions:**

The results of this study indicate that the range of motion of the CA has a significant impact on the magnitude of the PAPE response, and the greatest effect can be reached when the range of motion of the CA and the subsequent explosive task is similar.

## Introduction

The strength-power potentiation complex, recently named post-activation performance enhancement (PAPE), is an advanced training strategy used to acutely increase power performance [1]. It often involves a conditioning activity (CA), such as a heavy loaded bench press, followed by an explosive exercise with a similar movement structure, for example, the bench press throw (BPT) [2]. Performance enhancement often occurs 6–10 min after the CA and may be associated with increased muscle temperature, fiber water content, and muscle activation [3]. However, if a significant improvement is observed in the early stages after the CA (< 3 min), the contribution of the underlying mechanisms of the post-activation potentiation phenomenon cannot be ruled out, such as regulatory light chain phosphorylation, an increase in motor unit recruitment, and a change in the muscle fibers pennation angle [4].

Various upper and lower body exercises have been examined as a stimulus to induce PAPE [5–14]. These studies consistently indicate that the key variables of effective PAPE include volume, intensity, and the rest-interval between the CA and subsequent explosive task [15]. However, in addition to these variables, research indicates that the range of motion may also influence strength and power performance [15–17]. For example, Esformers and Bampauras [17] reported improvement in countermovement jump performance after both the quarter and parallel squat exercise as the CA. However, the parallel back squat was superior compared to the quarter back squat, whereas Mangus et al. [16] showed that part of the study participants increased their vertical jump after both, the parallel and quarter squats. In turn, Seitz and Haff [15] showed that shallow squat depths produced a considerably greater PAPE effect compared to deep squats. As suggested by the authors, this may be related to the induction of greater fatigue after deep squats than the shallow one [15]. Nevertheless, this effect is pronounced more in individuals with lower levels of muscle strength (< 1.75 relatives back squat strength) compared to their stronger counterparts (≥ 1.75) which seem to be more fatigue resistant [15]. Therefore, the optimal balance between fatigue and potentiation state is crucial to enhance subsequent performance. This will occur if potentiation exceeds fatigue; however, it will remain unchanged if fatigue and potentiation are at similar levels or decrease if fatigue dominates over potentiation [15]. A highly practical method that may serve as a sensitive indicator of neuromuscular fatigue includes the recording of velocity-loss during successive repetitions [18]. This solution may be useful when choosing the appropriate volume of the CA to maintain the optimal balance between potentiation and fatigue and contribute maximally to the PAPE effect. However, to date, only a few studies have investigated the use of velocity-loss control during a CA to optimize PAPE [19–22]. Tsoukos et al. [19, 20] showed that 10% velocity-loss (from the first repetition in a set) used during the bench press as a CA, led to a significant increase of the mean propulsive velocity during the subsequent BPT among resistance-trained males.

Moreover, since muscle activation varies through the range of motion, it can be speculated that this aspect may also influence the effectiveness of PAPE. For example, Krzysztofik et al. [23] showed that the cambered barbell (which allows for a greater range of motion than the standard one) was superior in activating the anterior deltoid muscle than the standard barbell during the bench press exercise. On the contrary, the standard barbell provided higher pectoralis major and triceps brachii long head activity. These muscles also play a significant role during throwing conditions [24], and the triceps shows the highest increase in activity compared to the bench press exercise. Therefore, it can be assumed that the standard barbell bench press will be a more effective CA than the cambered barbell bench press before a throwing performance. Considering that the greatest activity of the triceps is recorded in the final part of the barbell bench press [25], it was assumed that performing an inverted cambered barbell bench press as a CA will contribute to an even greater improvement in subsequent throwing performance.

Even though various squats depths have been examined, there is a lack of studies that evaluate this issue in case of upper-body PAPE complexes. Since the musculature of the upper and lower body differs significantly, it is not certain that the influence of the range of motion on the upper body PAPE effect will carry over to previous studies examining the lower body. Therefore, this study aimed to investigate the impact of different ranges of motion during the bench press exercise performed until 10% velocity-loss as a CA on subsequent BPT performance. We hypothesized that the PAPE effect would be visible after each applied CA, but the magnitude of improvement will be most significant after the reverse cambered barbell bench press.

## Materials and methods

### Experimental approach to the problem

**Fig. 1:**
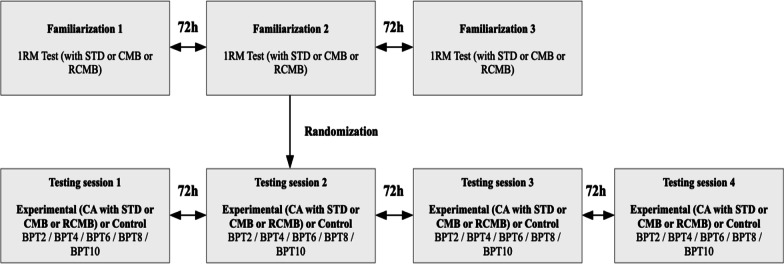
Study design. 1RM – one repetition maximum; CA—conditioning activity; STD—standard barbell conditioning activity; CMB—cambered barbell conditioning activity; RCMB—reverse cambered barbell conditioning activity; BPT—bench press throw

The participants took part in three familiarization sessions and four experimental sessions within three weeks. Separate familiarization sessions included the determination of one-repetition maximum (1RM) load for the standard (STD), cambered (CMB), and reverse cambered barbell (RCMB) in a randomized order (at least 72 h apart, using online software (randomization.org) and two sets of the BP performed with the corresponding barbell until 10% velocity-loss at 80%1RM. The experimental sessions were performed in randomized order (at least 72 h apart), where each participant performed a single set of the bench press at 80%1RM until mean barbell velocity dropped by 10% as the CA with a STD, a CMB, a RCMB barbell or a control condition in which the participants did not perform any CA (CTRL) (Fig. 1). The load of 80%1RM and 10% velocity-loss during the CA was chosen as earlier studies showed high effectiveness of this procedure in inducing the upper body PAPE effect [19, 20]. To assess the PAPE effect, single-sets of two repetitions of the BPT at 30%1RM (of the standard barbell) were performed 5 min before and after the CA in five-time points with 2 min rest intervals in between. Changes in peak power output (PP) and peak barbell velocity (PV) were evaluated.

### Subjects

Ten resistance-trained fitness specialists and personal trainers (age: 26 ± 3 years; body mass: 93.2 ± 9.4 kg; height: 181 ± 6 cm; experience in resistance training: 6.3 ± 2.4; standard bench press 1RM: 144.5 ± 26.2 kg and relative strength 1.54 ± 0.2 kg/body mass) participated in the study. The inclusion criteria were as follows: (i) free from neuromuscular and musculoskeletal disorders, (ii) at least three years’ experience in resistance training, (iii) relative standard bench press 1RM above 1.35 kg/body mass. Participants were excluded if they reported: (i) irregular participation in resistance training less than two times a week for the last six months, (ii) regular caffeine supplementation, (iii) had no experience in cambered barbell bench press. However, it should be emphasized that the participants had the most experience with a standard barbell bench press. Moreover, they were asked not to perform any additional resistance exercises at least 48 h before testing to avoid fatigue, maintain their usual dietary and sleep habits, and not to use any stimulants throughout the study. They were allowed to withdraw from the experiment at any moment and were informed about the benefits and potential risks of the study before providing their written informed consent for participation. The study protocol was approved by the Bioethics Committee for Scientific Research at the Academy of Physical Education in Katowice, Poland (10/2018) and performed according to the ethical standards of the Declaration of Helsinki, 2013 [26]. The sample size was calculated a priori based on a statistical power of 0.8, an effect size of 0.43, a correlation among repeated measures = 0.85, and an alpha level of p < 0.05, taking BPT performance as a reference variable [27]. A minimum sample size of 8 individuals was obtained (G*Power, Dusseldorf, Germany [version 3.1.9.2]).

### Procedures

#### Familiarization session and 1RM strength tests

Before the main experimental sessions, the 1RM bench press tests for each barbell type were performed in randomized order according to the recommendations proposed by Wilk et al. [28, 29]. The participants arrived in the laboratory at the same time of the day as the upcoming experimental sessions (in the morning between 9:00 and 11:00 am). The participants performed a standardized warm-up consisting of cycling on a stationary bike with an upper-body component for 5 min (Keiser M3i Total Body Trainer, Keiser Corporation, Fresno CA) at a resistance of approximately 100 W and cadence within 70–80 rpm; two circuits of 10 trunk rotations and side-bends; 10 internal, external and lateral arm swings; 10 bodyweight squats; 10 push-ups. Next, the participants performed 10, 8, and 4 repetitions at 30%, 50%, and 70% of their estimated 1RM with 2 min rest intervals. The first testing load was set to an estimated 80%1RM and was increased by 2.5–5 kg for each subsequent attempt until the participant couldn’t perform a lift with proper technique. A 5-min rest interval was allowed between 1RM attempts. If the participant failed, the last set was allowed with the load reduced by 2.5–5 kg. Hand placement on the barbell was set at 150% individual bi-acromial distance. All repetitions were performed without bouncing the bar off the chest, without intentionally pausing at the transition between the eccentric and concentric phases, and without raising the hips off the bench. The participants were instructed to perform each repetition with a two second duration of the eccentric phase (controlled by a metronome) and maximal velocity in the concentric phase of the movement [28, 29]. They were verbally motivated to make a maximum effort. The 1RM was defined as the highest load completed without any assistance from the spotters. All 1RM values were obtained within five attempts.

Following the 1RM test, all participants performed two sets of the bench press with a particular type of the barbell until a 10% mean velocity-loss at 80%1RM.

### Experimental sessions

The participants performed four different testing conditions, 72-h apart in a random order: a single set of a STD, CMB or RCMB barbell bench press at 80%1RM, with repetitions performed until mean movement velocity dropped by 10% or a control condition where participants did not perform the CA (CTRL). A single-set of two repetitions of the BPT on a Smith machine at 30%1RM (of the standard barbell) was performed 5 min before and after the CA at the following time points: 2, 4, 6, 8, 10 min. The participants began each condition by performing an identical warm-up as before the 1RM tests. The PAPE effect was evaluated by changes in PP and PV (by a linear position transducer) between baseline and post CA values during the BPT. The repetition with a higher value of PP and PV following the CA was kept for further analysis.

### Measurement of barbell velocity during the conditioning activity

Mean barbell velocity during the CA was controlled by the GymAware Powertool (Kinetic Performance Technology, Canberra, Australia), a linear position transducer. Previous research showed that this device provides reliable and valid kinematic data [30]. The device was placed on the floor directly under the barbell, and the external end of the cable was attached to the side of the barbell. The velocity of the barbell was recorded at 50 Hz. During the CA the participants were asked to perform each repetition with a constant duration of two seconds in the eccentric phase and as fast as possible during the concentric phase.

### Measurement of bench press throw performance

After the warm-up, the participants started the main trials. They performed a single set of two repetitions of the BPT with a maximal effort at 30% 1RM on a Smith machine as a baseline measurement. After a five min rest interval, they performed five sets of two repetitions of the BPT with two min rest intervals. During the CTRL condition, no CA was implemented. Before each trial, the participants were instructed to execute each repetition without bouncing the barbell off the chest, and without intentionally pausing at the transition between the phases. Hand placement on the barbell was set at 150% biacromial distance and was carefully replicated during each attempt. The PV and PP during the BPT were evaluated by a linear position transducer Tendo Power Analyzer (Tendo Sport Machines, Trencin, Slovakia). This device is a reliable system for measuring movement velocity and power output [31]. The between session intra-class correlation coefficient and coefficient of variation was 0.97 and 5.6% for PP, while for PV it was 0.88 and 2.7%, respectively. Due to the high inter-individual variability in the potentiation responses [32] and the individualized recovery time approach [5], the highest value obtained post-CA was retained for further analysis.

### Statistical analyses

All statistical analysis were performed using SPSS (version 25.0; SPSS, Inc., Chicago, IL, USA). The data is presented as means with standard deviations (± SD). Moreover, the 95% confidence intervals for mean values and relative differences (i.e. in percentages) between baseline and post-CA values are also provided. Statistical significance was set at p < 0.05. The normality of data distribution was checked using Shapiro–Wilk tests. The effects of the used CA on the dependent variables were examined by two-way repeated-measures ANOVA (4 conditions × 2 time points [baseline and best post-CA]). The effect size was determined by partial eta squared (η^2^). Partial eta squared values were classified as small (0.01 to 0.059), moderate (0.06 to 0.137) and large (> 0.137) [33]. When significant, pairwise comparisons were also conducted using a Bonferroni test. Moreover, the one-way ANOVA was performed to assess differences in 1RM and range of motion between STD, CMB and RCMB barbell conditions. The magnitude of mean differences was expressed with standardized effect sizes; thresholds for qualitative descriptors of Hedges g was interpreted as ≤ 0.20 “small”, 0.21–0.8 “medium”, and > 0.80 as “large” [33].

## Results

**Fig. 2:**
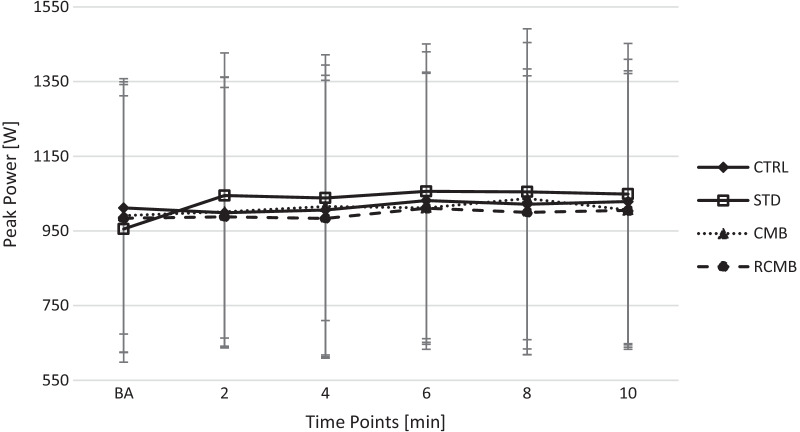
Time course of changes of peak power during the bench press throws

**Fig. 3:**
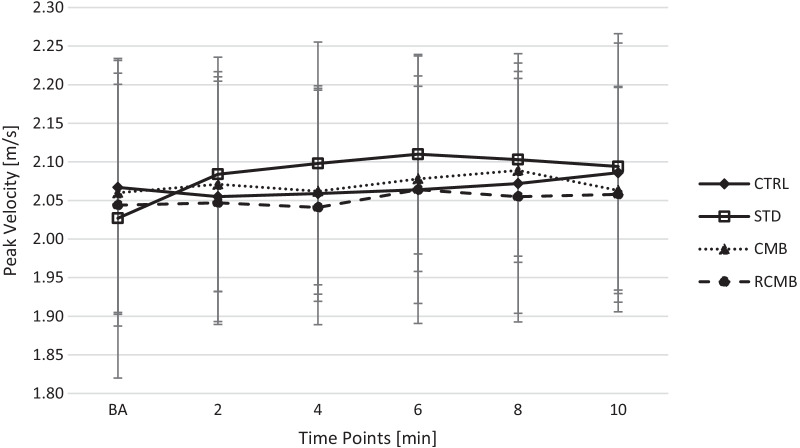
Time course of changes of peak velocity during the bench press throws

The one-way ANOVA indicated a significant difference in 1RM (144.5 ± 26, 138 ± 25, 153 ± 26 kg; p < 0.001) and range of motion (36 ± 3.6, 40 ± 3.7, 27 ± 3.8 cm; p < 0.001) for the STD, CMB and RCMB, respectively. The time course of changes of PP and PV during the BPT are presented in Figs. 2 and 3; respectively. The best post-CA performance was observed after 6.8 ± 2.5 min for CTRL condition, 5.2 ± 2.7 min for STD condition, 7.2 ± 2.7 min for the CMB condition, and 6.8 ± 1.9 min for the RCMB condition.

The Shapiro–Wilk tests indicated that the normality of the data wasn’t violated for PP and PV. The BPT performance after the CAs are presented in Table 1. The two-way ANOVA showed significant interaction for PP (p < 0.001; η^2^ = 0.556) and for PV (p = 0.001; η^2^ = 0.457). The post-hoc comparisons revealed a significant increase in post-CA PP and PV values during CTRL (p = 0.014 and p = 0.001), STD (p = 0.001 and p = 0.002), CMB (p = 0.005 and p = 0.011), and RCMB (p < 0.001 and p < 0.001) conditions compared to baseline. Furthermore, there were no significant differences between conditions.

**Table 1:** Baseline and best post-CA bench press throw performance

Condition	Baseline	Post-CA	ES	Relative effect (%)
Peak power (W)				
CTRL	1012 ± 338	1063 ± 384*	0.14	4.2 ± 4.5
STD	955 ± 357	1107 ± 405*	0.54	15.9 ± 9.4
CMB	991 ± 367	1068 ± 400*	0.19	7.3 ± 5.9
RCMB	984 ± 358	1036 ± 365*	0.14	5.6 ± 2.1
Peak velocity (m/s)				
CTRL	2.07 ± 0.16	2.12 ± 0.16*	0.30	2.3 ± 1.4
STD	2.03 ± 0.21	2.16 ± 0.13*	0.71	7.0 ± 6.2
CMB	2.06 ± 0.15	2.11 ± 0.12*	0.35	2.6 ± 2.8
RCMB	2.04 ± 0.16	2.09 ± 0.15*	0.31	2.2 ± 1.3

Results are mean ± SD; *significant difference in comparison to baseline p < 0.05; *CA* conditioning activity; *CTRL* control condition; *STD* standard barbell condition; *CMB* cambered barbell condition; *RCMB* reverse cambered barbell condition; *ES* Hedges g effect size

## Discussion

The main finding of this study was that the STD bench press as a CA led to the greatest enhancement of BPT performance (+ 15.9%; ES = 0.54 in PP and + 7.0%; ES = 0.71 in PV) within the examined conditions (Table 1). Contrary to the initial hypothesis, the RCMB bench press as a CA was the least effective. It led to a slight improvement in efficiency (+ 5.6%; ES = 0.14 in PP and + 2.2%; ES = 0.31 in PV), comparable with the CTRL condition (+ 4.2%; ES = 0.14 in PP and + 2.3%; ES = 0.3 in PV). Thus, these results indicate that the range of motion has a significant impact on the magnitude of the PAPE effect. This suggests that to acutely improve upper-body explosive performance, is it recommended to prescribe a CA with a similar movement pattern that will follow in the subsequent explosive task.

To our knowledge, despite that many studies evaluated different back squat depths as a CA [34–36], only two studies directly compared its effectiveness in enhancing performance [16, 17]. While in the case of upper body enhancement complexes, the most commonly used CA is the STD bench press [15, 27, 37], and to date, no study has considered the impact of a varied range of motion on PAPE. It should be noted that during the bench press exercise performed with a standard type barbell, the range of motion is restricted by the barbell touching the chest, while the prime movers are not going through their full physiological range of motion [38]. Using a cambered barbell, which is U-shaped and creates additional space for the chest, enables the movement to a lower end position than a standard barbell [39]. In turn, reversing this barbell allows for a significantly shorter range of motion.

The results of this study are partially in line with the findings of Esformes and Bampauras [17], as the use of the STD bench press as a CA led to a greater improvement in BPT performance than the RCMB bench press. We also evaluated the effectiveness of the CMB bench press as a CA, in which the range of motion was the greatest. However in this case, a STD bench press as the CA proved to elicit greater PAPE magnitude. The analysis of baseline and the highest value obtained post-CA irrespective of the rest interval also showed a similar pattern of performance enhancement, with the highest one obtained after a standard bar CA (+ 7.0–15.9%; ES = 0.54–0.71), then the CMB (+ 2.6–7.3%; ES = 0.19–0.35) and at least after the RCMB bench press exercise (+ 2.2–5.6%; ES = 0.14–0.31). It is worth noting that the level of performance enhancement during the RCMB bench press was similar to the CTRL condition (+ 2.3–4.2%; ES = 0.14–0.3), which indicates that subsequent sets of BPT may also induce a low magnitude of the PAPE effect. On the other hand, a previous study by Nibali et al. [40] showed insignificant differences between continuous and intermittent measurements post-CA. However, it has to be mentioned that in the current study, 2-min rest intervals between consecutive post-CA were used, while in the study of Nibali et al. [40], it was 4 min. Therefore, future studies should consider that repeatedly performed post-CA evaluations may inadvertently act as a CA, affecting the magnitude of fatigue and/or potentiation. As our participants were highly trained and familiar with the CMB bench press, considering that fatigue was individually controlled by the velocity loss under all conditions, it seems that the influence of excessive fatigue can be ruled out. This was also confirmed by the lowest enhancement of the BPT following the RCMB CA. Therefore, these results appear to be related to the level of induced potentiation, not to the level of fatigue.

We cannot indicate which physiological mechanisms contributed to the observed performance improvement. Based on previous research, we can speculate that it is related, as indicated by Tsoukos et al. [20] to the neural mechanisms (e.g., increased recruitment of motor units or increased excitability of motoneurons) instead of other suggested mechanisms such as increased: regulatory myosin light-chain phosphorylation, muscle temperature or intramuscular fluid accumulation [3]. The volume in each of the CAs used was low; thus, the effect of temperature or fluid accumulation in the muscles seems unlikely. This may be confirmed by the study of Weigert et al. [41] which showed that a single set of resistance exercise did not lead to a significant increase in muscle temperature. Furthermore, given that in most cases, the peak performance occurred 6 min after the CA, the contribution of increased myosin light-chain phosphorylation in performance enhancement seems to be insignificant due to its short duration following the CA (~ 28 s and a small performance enhancement effect observed within ~ 5 min) [42–45]. Therefore, the most likely explanation for the performance enhancement may relate to the similarities of the muscular activation between the CA and the BPT. We can assume that the RCMB bench press led to the greatest activation of the triceps brachii, but also the smallest of the remaining muscles involved in the BPT movement such as the pectoralis major and anterior deltoid [46]. As a result, the improvement in the BPT performance under this condition was small. In turn, a slightly better effect was recorded after the CMB bench press CA, perhaps because the barbell enables a greater stretch of the chest and shoulder muscles, thus providing additional activation of the above-mentioned muscles compared to the RCMB barbell bench press. On the other hand, the STD bench press CA turned out to be optimal, perhaps due to the similarity of the movement to the BPT, thus probably providing an adequate level and similar pattern of muscle excitation. This may also explain parallel squats' high effectiveness in jump height enhancement due to the similarity in preferred knee flexion degree during vertical jump tests [17, 47]. Therefore, when we summarize the results of our investigation and the studies that examined various squat depths, it seems that when a range of motion exceeds a certain threshold, the subsequent performance shows no further improvement, or it may even decrease [15]. The results of this study indicate that the range of motion affects the PAPE response and confirm the statement of similarity requirements between the CA and the subsequent explosive activity to obtain the greatest enhancement in performance.

In addition to its strengths, the present study has several limitations which need to be addressed: (1) only male, strength trained participants took part in this study. Therefore, bearing in mind high inter-individual variability in the PAPE responses [32] and also possible differences in magnitude of PAPE between post-CA activities [15], caution is needed when extrapolating these results to other populations and conditions; (2) in each of the conditions used, the participants performed only a single set, at one intensity (80%1RM) and with one predetermined movement velocity-loss (by 10%), (3) we did not evaluate any of the physiological mechanisms which underlie the PAPE effect. Therefore, no definite conclusions can be derived from the study, besides the fact that the results of this study indicate that the range of motion affects the PAPE response and confirm the statement of similarity requirements between the CA and the subsequent explosive activity.

## Conclusions

This study demonstrated that the range of motion of the CA has a significant impact on the magnitude of the PAPE response. Therefore, individuals seeking to acutely enhance their upper-body explosive performance should consider that the CA and subsequent explosive task should be as similar as possible in terms of movement pattern to maximize the PAPE effect.

## Data Availability

The datasets analysed during the current study are available at https://data.mendeley.com/datasets/7h88r9byb4/1.

## Abbreviations

PAPE: Post-activation performance enhancement
CA: Conditioning activity
BPT: Bench press throw
1RM: One-repetition maximum
STD: Standard
CMB: Cambered barbell
RCMB: Reverse cambered barbell
CTRL: Control
PP: Peak power
PV: Peak velocity

## References

[1] Suchomel TJ, Lamont HS, Moir GL (2016). Understanding vertical jump potentiation: a deterministic model. Sports Med.

[2] Krzysztofik M, Wilk M, Filip A, Zmijewski P, Zajac A, Tufano JJ (2020). Can post-activation performance enhancement (PAPE) improve resistance training volume during the bench press exercise?. Int J Environ Res Public Health.

[3] Blazevich AJ, Babault N (2019). Post-activation potentiation versus post-activation performance enhancement in humans: historical perspective, underlying mechanisms, and current issues. Front Physiol.

[4] Tillin NA, Bishop D (2009). Factors modulating post-activation potentiation and its effect on performance of subsequent explosive activities. Sports Med.

[5] Gołaś A, Maszczyk A, Zajac A, Mikołajec K, Stastny P (2016). Optimizing post activation potentiation for explosive activities in competitive sports. J Hum Kinet.

[6] Tsoukos A, Bogdanis GC, Terzis G, Veligekas P (2016). Acute improvement of vertical jump performance after isometric squats depends on knee angle and vertical jumping ability. J Strength Cond Res.

[7] Ulrich G, Parstorfer M (2017). Effects of plyometric versus concentric and eccentric conditioning contractions on upper-body postactivation potentiation. Int J Sports Physiol Perform.

[8] Beato M, Bigby AEJ, De Keijzer KL, Nakamura FY, Coratella G, McErlain-Naylor SA (2019). Post-activation potentiation effect of eccentric overload and traditional weightlifting exercise on jumping and sprinting performance in male athletes. PLoS ONE.

[9] Gepfert M, Golas A, Zajac T, Krzysztofik M (2020). The use of different modes of post-activation potentiation (PAP) for enhancing speed of the slide-step in basketball players. Int J Environ Res Public Health.

[10] Matusiński A, Pietraszewski P, Krzysztofik M, Gołaś A (2021). The effects of resisted post-activation sprint performance enhancement in elite female sprinters. Front Physiol.

[11] Krzysztofik M, Kalinowski R, Filip-Stachnik A, Wilk M, Zajac A (2021). The effects of plyometric conditioning exercises on volleyball performance with self-selected rest intervals. Appl Sci.

[12] Krzysztofik M, Wilk M (2020). The effects of plyometric conditioning on post-activation bench press performance. J Hum Kinet.

[13] Krzysztofik M, Wilk M, Golas A, Lockie RG, Maszczyk A, Zajac A (2020). Does eccentric-only and concentric-only activation increase power output?. Med Sci Sports Exerc.

[14] Krzysztofik M, Wilk M, Lockie RG, Golas A, Zajac A, Bogdanis GC. Postactivation performance enhancement of concentric bench press throw after eccentric-only conditioning exercise. J Strength Cond Res. 2020. https://journals.lww.com/nsca-jscr/abstract/9000/postactivation_performance_enhancement_of.94265.aspx. 10.1519/JSC.0000000000003802 32826834

[15] Seitz LB, Haff GG (2016). Factors modulating post-activation potentiation of jump, sprint, throw, and upper-body ballistic performances: a systematic review with meta-analysis. Sports Med.

[16] Mangus BC, Takahashi M, Mercer JA, Holcomb WR, McWhorter JW, Sanchez R (2006). Investigation of vertical jump performance after completing heavy squat exercises. J Strength Cond Res.

[17] Esformes JI, Bampouras TM (2013). Effect of Back Squat Depth on Lower-Body Postactivation Potentiation. J Strength Cond Res.

[18] Rodríguez-Rosell D, Yáñez-García JM, Mora-Custodio R, Pareja-Blanco F, Ravelo-García AG, Ribas-Serna J (2020). Velocity-based resistance training: impact of velocity loss in the set on neuromuscular performance and hormonal response. Appl Physiol Nutr Metab.

[19] Tsoukos A, Brown LE, Terzis G, Veligekas P, Bogdanis GC (2020). Potentiation of bench press throw performance using a heavy load and velocity-based repetition control. J Strength Cond Res.

[20] Tsoukos A, Brown LE, Veligekas P, Terzis G, Bogdanis GC (2019). Postactivation potentiation of bench press throw performance using velocity-based conditioning protocols with low and moderate loads. J Hum Kinet.

[21] Krzysztofik M, Matykiewicz P, Celebanska D, Jarosz J, Gawel E, Zwierzchowska A (2021). The acute post-activation performance enhancement of the bench press throw in disabled sitting Volleyball athletes. Int J Environ Res Public Health.

[22] Krzysztofik M, Kalinowski R, Trybulski R, Filip-Stachnik A, Stastny P (2021). Enhancement of countermovement jump performance using a heavy load with velocity-loss repetition control in female Volleyball players. Int J Environ Res Public Health.

[23] Krzysztofik M, Golas A, Wilk M, Stastny P, Lockie RG, Zajac A (2020). A comparison of muscle activity between the cambered and standard bar during the bench press exercise. Front Physiol.

[24] Newton RU, Kraemer WJ, Häkkinen K, Humphries BJ, Murphy AJ (1996). Kinematics, kinetics, and muscle activation during explosive upper body movements. J Appl Biomech.

[25] van den Tillaar R, Saeterbakken A, Ettema G (2012). Is the occurrence of the sticking region the result of diminishing potentiation in bench press?. J Sports Sci.

[26] World Medical Association Declaration of Helsinki (2013). Ethical principles for medical research involving human subjects. JAMA.

[27] Krzysztofik M, Wilk M, Stastny P, Golas A (2021). Post-activation performance enhancement in the bench press throw: a systematic review and meta-analysis. Front Physiol.

[28] Wilk M, Golas A, Zmijewski P, Krzysztofik M, Filip A, Coso JD (2020). The effects of the movement tempo on the one-repetition maximum bench press results. J Hum Kinet.

[29] Wilk M, Gepfert M, Krzysztofik M, Mostowik A, Filip A, Hajduk G (2020). Impact of duration of eccentric movement in the one-repetition maximum test result in the bench press among women. J Sports Sci Med.

[30] Banyard HG, Nosaka K, Sato K, Haff GG (2017). Validity of various methods for determining velocity, force, and power in the back squat. Int J Sports Physiol Perform.

[31] Garnacho-Castaño MV, López-Lastra S, Maté-Muñoz JL (2015). Reliability and validity assessment of a linear position transducer. J Sports Sci Med.

[32] Boullosa D, Beato M, Dello Iacono A, Cuenca-Fernández F, Doma K, Schumann M (2020). A new taxonomy for post-activation potentiation in sport. Int J Sports Physiol Perform.

[33] Cohen J (2013). Statistical Power Analysis for the Behavioral Sciences.

[34] Kilduff LP, Bevan HR, Kingsley MIC, Owen NJ, Bennett MA, Bunce PJ (2007). Postactivation potentiation in professional rugby players: optimal recovery. J Strength Cond Res.

[35] Bauer P, Sansone P, Mitter B, Makivic B, Seitz LB, Tschan H (2019). Acute effects of back squats on countermovement jump performance across multiple sets of a contrast training protocol in resistance-trained men. J Strength Cond Res.

[36] Timon R, Allemano S, Camacho-Cardeñosa M, Camacho-Cardeñosa A, Martinez-Guardado I, Olcina G (2019). Post-activation potentiation on squat jump following two different protocols: traditional Vs. Inertial Flywheel J Hum Kinet.

[37] Wilson JM, Duncan NM, Marin PJ, Brown LE, Loenneke JP, Wilson SMC (2013). Meta-analysis of postactivation potentiation and power: effects of conditioning activity, volume, gender, rest periods, and training status. J Strength Cond Res.

[38] Krzysztofik M, Zajac A, Żmijewski P, Wilk M (2020). Can the cambered bar enhance acute performance in the bench press exercise?. Front Physiol.

[39] Krzysztofik M, Matykiewicz P, Filip-Stachnik A, Humińska-Lisowska K, Rzeszutko-Bełzowska A, Wilk M (2021). Range of motion of resistance exercise affects the number of performed repetitions but not a time under tension. Sci Rep.

[40] Nibali ML, Chapman DW, Robergs RA, Drinkwater EJ (2015). Considerations for determining the time course of post-activation potentiation. Appl Physiol Nutr Metab.

[41] Weigert M, Nitzsche N, Kunert F, Lösch C, Baumgärtel L, Schulz H (2018). Acute exercise-associated skin surface temperature changes after resistance training with different exercise intensities. Int J Kinesiol Sports Sci.

[42] Vandervoort AA, Quinlan J, McComas AJ (1983). Twitch potentiation after voluntary contraction. Exp Neurol.

[43] O’Leary DD, Hope K, Sale DG (1997). Posttetanic potentiation of human dorsiflexors. J Appl Physiol.

[44] Hamada T, Sale DG, MacDougall JD, Tarnopolsky MA (2000). Postactivation potentiation, fiber type, and twitch contraction time in human knee extensor muscles. J Appl Physiol.

[45] MacIntosh BR, Willis JC (2000). Force-frequency relationship and potentiation in mammalian skeletal muscle. J Appl Physiol.

[46] Stastny P, Gołaś A, Blazek D, Maszczyk A, Wilk M, Pietraszewski P (2017). A systematic review of surface electromyography analyses of the bench press movement task. PLoS ONE.

[47] Domire ZJ, Challis JH (2007). The influence of squat depth on maximal vertical jump performance. J Sports Sci.

